# Factors Associated with Appointment Non-Adherence among African-Americans with Severe, Poorly Controlled Hypertension

**DOI:** 10.1371/journal.pone.0103090

**Published:** 2014-08-14

**Authors:** Chike C. Nwabuo, Sydney Morss Dy, Kristina Weeks, J. Hunter Young

**Affiliations:** 1 Division of Cardiology, Department of Medicine, Johns Hopkins Medical Institutions, Baltimore, MD, United States of America; 2 Department of Medicine, Johns Hopkins Medical Institutions, Baltimore, MD, United States of America; 3 Department of Health Policy and Management, Johns Hopkins Bloomberg School of Public Health, MD, United States of America; 4 Department of Anesthesiology and Critical Care Medicine, Johns Hopkins Medical Institutions, Baltimore, MD, United States of America; University of Sao Paulo Medical School, Brazil

## Abstract

**Background:**

Missed appointments are associated with an increased risk of hospitalization and mortality. Despite its widespread prevalence, little data exists regarding factors related to appointment non-adherence among hypertensive African-Americans.

**Objective:**

To investigate factors associated with appointment non-adherence among African-Americans with severe, poorly controlled hypertension.

**Design and Participants:**

A cross-sectional survey of 185 African-Americans admitted to an urban medical center in Maryland, with severe, poorly controlled hypertension from 1999–2004. Categorical and continuous variables were compared using chi-square and t-tests. Adjusted multivariable logistic regression was used to assess correlates of appointment non-adherence.

**Main Outcome Measures:**

Appointment non-adherence was the primary outcome and was defined as patient-report of missing greater than 3 appointments out of 10 during their lifetime.

**Results:**

Twenty percent of participants (n = 37) reported missing more than 30% of their appointments. Patient characteristics independently associated with a higher odds of appointment non-adherence included not finishing high school (Odds ratio [OR] = 3.23 95% confidence interval [CI] (1.33–7.69), hypertension knowledge ([OR] = 1.20 95% CI: 1.01–1.42), lack of insurance ([OR] = 6.02 95% CI: 1.83–19.88), insurance with no medication coverage ([OR] = 5.08 95% CI: 1.05–24.63), cost of discharge medications ([OR] = 1.20 95% CI: 1.01–1.42), belief that anti-hypertensive medications do not work ([OR] = 3.67 95% CI: 1.16–11.7), experience of side effects ([OR] = 3.63 95% CI: 1.24–10.62), medication non-adherence ([OR] = 11.31 95% CI: 3.87–33.10). Substance abuse was not associated with appointment non-adherence ([OR] = 1.05 95% CI: 0.43–2.57).

**Conclusions:**

Appointment non-adherence among African-Americans with poorly controlled hypertension was associated with many markers of inadequate access to healthcare, knowledge, attitudes and beliefs.

## Introduction

Physician appointments provide an important avenue for blood pressure control through patient education, medication titration and early detection of complications. Appointment non-adherence constitutes an obstacle to the provision of adequate patient care and may be associated with poor control of chronic illness [1]–[4], increased risk of hospitalization [5], [6], reduced clinic efficiency [7], and mortality [8]. This is especially true among minorities in the United States [9], and among individuals with chronic conditions such as hypertension [10]. African-Americans in low resource communities experience greater difficulty achieving adequate blood pressure compared to other Americans [11], [12], and also have comparatively greater difficulty with adherence [13], [14] [15]. These differences may contribute to observed ethnic disparities in mortality [16].

Several interventions have been utilized in improving appointment adherence [17]–[20]. In spite of the availability of these interventions, appointment non-adherence remains a burden to the provision of quality healthcare. Designing effective and socially appropriate interventions that improve appointment adherence require a better understanding of the factors associated with this behavior. Despite its importance, little data exists regarding factors related to appointment non-adherence among hypertensive African-Americans [8], [21], [22]. Therefore, to fill this gap, this study examined factors associated with patient-reported appointment adherence among African-Americans with severe, poorly controlled hypertension, using data from the Inner City Hypertension and Body Organ Damage (ICHABOD) - a cross-sectional survey of urban African-Americans hospitalized with severe, poorly controlled hypertension.

## Methods

### Study Design, Setting and Participants

Baseline cross-sectional survey data was analyzed from the ICHABOD study- a cross-sectional survey of urban African-Americans with severe, poorly controlled hypertension described previously [23], [24]. Study was approved by the Johns Hopkins Medicine Institutional Review Board and all study participants provided written informed consent.

### Data Source

Source population included all patients admitted to a large urban medical center in Baltimore, MD, from August 1999 to June 2001 and from February 2002 to December 2004. Utilizing an automatic oscillatory device (Dinamap), we identified patients with severe hypertension, defined as a systolic blood pressure (SBP)≥180 mmHg and/or a diastolic blood pressure (SBP)≥110 mmHg, on two separate occasions. We excluded study participants with the following characteristics 1) Hypertension due to known secondary cause 2) Newly diagnosed cases of hypertension 3) Age <18 years 4) Non-residence in Baltimore city 5) Ethnicity other than African-American. Of the 485 patients identified with severe, poorly controlled hypertension, 196 (40%) were excluded because they had secondary causes of hypertension, were newly diagnosed cases of hypertension, or did not give informed consent. Twenty-one (4%) participants died in the hospital before being enrolled into the study. Of the 269 remaining patients, 84 (31.8%) patients refused, withdrew, never completed the questionnaire, or were discharged prior to contact. Thus, of the eligible patients, 185 were included in this analysis, yielding an overall response rate of 68.2%.

### Data collection

Trained interviewers administered a structured questionnaire upon admission. The questionnaire was administered in a non-judgmental manner to optimize patient disclosure. Interviewers reviewed the admission history and physical examination; patients discharge notes were also reviewed. Study questionnaire was adapted from previously validated instruments, modeled after those used in trials conducted in inner-city populations to improve the control of hypertension and diabetes [25]–[28], and further refined through a pilot [23], [24]. The questionnaire assessed hypertension history, substance abuse, socio-economic factors, co-morbidities, disease severity, medication and appointment adherence patterns, reasons for non-adherence (if non-adherence was reported), access to care, attitudes, beliefs, and knowledge of hypertension and its consequences. Other measurements included insurance coverage (self-reported combined with medical records and hospital billing data) and self-reported difficulty of obtaining medications. For participants who missed appointments, survey questions addressed perceived barriers to appointment attendance.

### Measures/Definitions

#### Appointment Non-adherence

**Figure 1:**
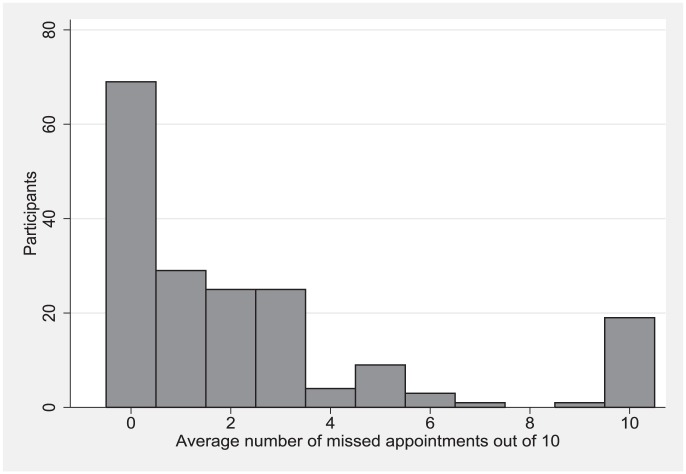
Distribution of missed appointments among 185 African-Americans admitted to an urban hospital to an urban hospital with severe hypertension. Each participant was asked “Out of every 10 appointments, on average how many do you miss?

Appointment Non-adherence was defined as the tendency to miss greater than 3 appointments out of 10, when asked the question “Out of every 10 appointments, on average how many do you miss?” This was defined using the frequency distribution of missed appointments (Figure 1), as well as cutoffs from previous adherence studies [2], [21].

#### Medication non-adherence

We assessed medication adherence in two different dimensions, which measure distinct aspects of adherence behaviors; pill-taking behavior and prescription refill behavior.

Medication non-adherence (with regards to pill taking behavior) was defined as the tendency to miss one or more pills per week. This was assessed by the question, “On average, how many times a week do you miss taking your blood pressure pills?”

Medication non-adherence (with regards to prescription refill behavior) was defined as the tendency of running out of medication, for at least a day or two, 3 or more times per year. This was assessed by the question, “On average, how many times a year do you run out of your pills for at least a day or two?”

#### Substance abuse

Substance abuse was measured using a combination of self-report and biochemical tests of urine. If participants reported using a drug during the 2 weeks prior to admission or if the urine toxicology test was positive for that drug, the participant was considered an active user of the drug.

#### Disease Severity

Disease severity was quantified using the risk of mortality score and disease complexity score, derived from 3M's All Patient Refined Diagnostic Related Groups (APR-DRG) scoring system, V20 [29]. The APR-DRG risk of mortality scores and disease complexity scores have 4 categories on an ordinal scale (1 = minor, 2 = moderate, 3 = major, and 4 = extreme) and each score measures different aspects of disease severity [30]. APR-DRG risk of mortality and disease complexity categories 3 and 4 were combined due to the small number of participants in the highest risk groups. Both were included for risk adjustment as categorical variables to match participants of similar disease severity [31]. Co-morbid illness was assessed through self-report, chart review, and the discharge diagnoses (coded using the International Classification of Disease, Ninth Revision, Clinical Modification (ICD-9-CM)).

### Data Analysis

Descriptive frequencies of demographic, socio-economic characteristics and medication non-adherence were assessed overall and by category of appointment adherence. Categorical variables were compared using chi-squared statistics for frequency and proportions. Continuous variables were presented as means and compared using t-tests. Significance level was set at P<.05 for all analyses.

To investigate correlates of appointment non-adherence, multivariable logistic regression analyses were performed assessing for age, sex, education, employment, insurance, substance abuse, depression, disease complexity and mortality risk, hypertension knowledge, side effects belief medications don't work, medication non-adherence, cost of discharge medications.

In order to account for missing data (numbers of missing responses in each variable are described in Table 1), multiple imputation by chained equations (MICE) method in STATA was applied using the missing at random (MAR) assumption. Multivariable logistic regressions were then performed in each of the 5 imputed datasets and combined using standardized statistical methods for multiple imputation. A sensitivity analysis was carried out to assess the impact of imputation on these estimates by complete-case analysis of study participants. These results were similar and thus not reported. All analyses were performed with Stata version 12 (StataCorp 2009, College Station, TX USA).

**Table 1:** Characteristics of 185 African-Americans admitted to an urban hospital with severe hypertension, poorly controlled hypertension.

Characteristics	Overall	Appointment adherent	Appointment non-adherent	P-value
	N (SD or %)	N (SD or %)	N (SD or %)	
N	185	148(80%)	37(20%)	
**Demographics**				
Age (years)	51.1(12.2)	50.9(11.9)	51.9(13.2)	0.66
Female (%)	101(54.6)	81(54.7)	20(54.1)	0.94
Completed high school	97(52.7)	85(57.8)	12(32.4)	**<0.01**
Employed full or part-time	54(29.2)	45(30.4)	9(24.3)	0.47
Currently married (%)	47(25.4)	39(26.4)	8(21.6)	0.55
**Disease characteristics**				
Mean Systolic blood pressure(mmHg)	201.8(18.7)	200.7(18.6)	205.95(18.8)	0.13
Mean Diastolic blood pressure(mmHg)	122.7(13.6)	121.64(13.3)	127.1(14.1)	**0.03**
**Comorbidities**				
Diabetes	54(29.2)	47(31.8)	7(18.9)	0.12
End Stage Renal Disease	28(15.1)	22(14.9)	6(16.2)	0.84
HIV	10(5.4)	9(6.1)	1(2.7)	0.42
Depression	12(6.5)	11(7.43)	1(2.7)	0.30
**Substance use**				
Current heavy alcohol use (%)	23(12.4)	16(10.8)	7(18.9)	0.18
Current heroin and/or cocaine use (%)	58(31.4)	45(30.4)	13(35.1)	0.58
**APR-DRG Risk of Mortality**				
Level 1 “Minor”	78(42.2)	62(41.9)	169(43.2)	Ref
Level 2 “Moderate”	67(36.2)	54(36.5)	13(35.1)	0.87
Level 3/4 “Major” and “Extreme”	40(33.9)	32(34.0)	8(33.3)	0.95
**Knowledge, Attitude & beliefs**				
Inadequate hypertension knowledge(<80% correct)^*^	49(26.5)	34(23.0)	15(40.5)	**0.03**
Experience side effects	32(18.6)	23(16.1)	9(31.0)	**0.059**
Difficulty paying for medication	95(51.9)	71(48.0)	24(68.6)	**0.03**
Belief that medications don't work	25(14.6)	17(12.0)	8(27.6)	**0.03**
**Insurance Status**				
Full medication coverage	52 (28.1)	47 (31.8)	5 (13.5)	Ref
Medication coverage with copays	56 (30.3)	52 (35.1)	4 (10.8)	0.64
No Medication coverage	15(8.1)	10 (6.8)	5 (13.5	**0.02**
No Insurance	62 (33.5)	39 (26.4)	23 (62.2)	**<0.01**
**Medication non-adherence**				
Medication non-adherence (Pill taking behavior)	61(36.3)	45(31.5)	16(64.0)	**<0.01**
Medication non-adherence (Prescription refill)	58(31.4)	36(24.3)	22(59.5)	**<0.01**

16 participants (7.6%) were missing data for the variable ‘medications don't work’, 13 participants (7.0%) were missing data for the variable ‘experience side effects’, 12 participants (6.5%) were missing data for the variable can't afford medications, 5 participants (2.7%) were missing data for the variable ‘cost of discharge medications’, 1 participant (<1%) was missing high school status.

## Results

A total of 185 African-Americans with severe, poorly controlled hypertension were enrolled into the study. Twenty percent of participants (n = 37) missed more than 30% of their appointments. The median age at study entry was 48.4 years (interquartile range [IQR], 43.1–57.9). Among study participants, 54.6% (n = 101) were women, 52.6% (n = 97) completed high school or its equivalent, about one-quarter (n = 47) were married, and only 29% (n = 54) were employed either full or part-time, and more than one-third of participants (n = 62) were uninsured. Self-reported barriers to healthcare access included trouble affording doctor visits (51.7%), forgetfulness (30.2%), transportation (30.2%), trouble getting through at the doctor's office (19.8%), and feeling that doctor appointments are not helpful (10.3%).

The APR-DRG Risk of Mortality score was greater than level 1 in 57.8% of study participants and mean SBP and DBP were 201.9 (SD 18.6) and 122.3 (SD 12.9) mm Hg respectively. Over a quarter (n = 49) answered fewer than 80% of a set of true/false hypertension knowledge questions correctly. About half of the study participants reported difficulty paying for medication, 18.6% (n = 32) experienced side effects, and 14.6% (n = 25) believed that anti-hypertensive medications do not work. Almost a third (n = 58) reported having run out of their medications for a day or more at least 3 times per year and 36.3% (n = 61) reported missing at least 1 dose of medication in a typical week.

As illustrated in Table 1, the distribution of unadjusted patient characteristics varied across the categories of appointment keeping. Appointment non-adherence was associated with completing high school, higher diastolic blood pressure, poor hypertension knowledge, experience of side-effects, self-reported difficulty paying for medication, belief that medications do not work lack of insurance, and medication non-adherence.

Table 2 presents results of a multivariable logistic regression analysis demonstrating the association of several factors with appointment non-adherence after adjustment for potential confounders. Patient characteristics associated with a higher odds of appointment non-adherence included not finishing high school ([OR] = 3.23 95% CI (1.33–7.69), lack of insurance ([OR] = 6.02, 95% CI: 1.83–19.88), insurance without medication coverage ([OR] = 5.08, 95% CI: 1.05–24.63), higher cost of discharge medications ([OR] = 1.20 95% CI: 1.01–1.42), poor hypertension knowledge ([OR] = 2.39 95% CI: 1.01–5.78), experience of side effects ([OR] = 3.63 95% CI: 1.24–10.62), belief that anti-hypertensive medications do not work ([OR] = 3.67 95% CI: 1.16–11.67), medication non-adherence (pill taking behavior [OR] = 11.31 95% CI: 3.87–33.10) and prescription refill non-adherence ([OR] = 3.50 95% CI: 1.45–8.44).

**Table 2:** Adjusted odd ratios for associations of appointment non-adherence among 185 African-Americans admitted to an urban hospital with severe, poorly controlled hypertension.

Variable	Adjusted odds ratio (95% CI)	P - value
Age (per 10 yrs)	1.01 (0.97–1.05)	0.70
Men (Women are reference)	0.91 (0.39–2.11)	0.83
Not Finishing high school	**3.23 (1.33–7.69)**	**<0.01**
Employed full or part-time	1.06 (0.38–2.86)	0.91
Heroin and/or Cocaine use	1.05 (0.43–2.57)	0.92
Depression	0.99 (0.10–9.82)	0.99
**Mortality risk category**		
Level 1 “Minor”	Reference	
Level 2 “Moderate”	0.61 (0.20–1.83)	0.38
Level 3/4 “Major” and “Extreme”	0.54 (0.12–2.34)	0.41
**Disease complexity category**		
Level 1 “Minor”	Reference	
Level 2 “Moderate”	1.16 (0.26–5.17)	0.81
Level 3/4 “Major” and “Extreme”	3.14 (0.53–18.46)	0.20
**Access to care**		
Cost of discharge medications (per $10)	**1.20 (1.01–1.42)**	**0.04**
**Insurance Status**		
Full Medication coverage	Reference	
Medication Coverage with copay	0.72 (0.16–3.25)	0.67
No Medication Coverage	**5.08 (1.05–24.63)**	**0.04**
No Insurance	**6.03 (1.83–19.88)**	**<0.01**
**Knowledge, Attitudes & Beliefs**		
Hypertension knowledge(<80% correct)	**2.39 (1.01–5.78)**	**0.05**
Experience side effects	**3.63(1.24–10.62)**	**0.03**
Belief that medications don't work	**3.67 (1.16–11.67)**	**0.03**
**Medication non-adherence**		
Medication non-adherence (Pill taking behavior)	**11.31 (3.87–33.10)**	**<0.01**
Medication non-adherence (Prescription refill behavior)	**3.50 (1.45–8.44)**	**<0.01**

Model adjusted for age, gender, education, employment status, disease complexity, mortality risk, depression, substance abuse (heroin and/or cocaine use), and insurance Status, **bold indicates P<0.05.**

In contrast, this study did not identify any statistically significant association between appointment non-adherence and age ([OR] = 1.01 95% CI: 0.97–1.05), sex (men, [OR] = 0.91 95% CI: 0.39–2.11), unemployment ([OR] = 1.06 95% CI: 0.38–2.86), substance abuse ([OR] = 1.05 95% CI 0.43–2.57), and depression ([OR] = 0.99 95% CI: 0.10–9.82).

## Discussion

In this cross-sectional study of urban African-Americans with severe, poorly controlled hypertension, factors associated with appointment non-adherence included lack of health insurance, insurance without medication coverage, inadequate hypertension knowledge, experience of side effects, not finishing high school, high cost of discharge medications, belief that medications do not work, and medication non-adherence. Perceived self-reported barriers to appointment attendance included forgetfulness, transportation, trouble getting through at the doctor's office, and feeling that appointments are not helpful.

This study highlighted several factors related to appointment non-adherence among urban African-Americans with severe, poorly controlled hypertension. Uninsured individuals were more likely to exhibit appointment non-adherence compared to those with full medication coverage. In addition, among insured individuals, those with no medication coverage were also more likely to exhibit appointment non-adherence. Our study findings also indicated that high cost of discharge medications were associated with appointment non-adherence. Previous research has shown no association between insurance status, reported difficulty affording medications and antihypertensive discharge regimen costs [24]. These relationships demonstrate the positive impact of insurance with medication coverage on appointment keeping behavior and underscore the need for provision of more transparent information regarding prescription costs. These findings are consistent with studies that have demonstrated a relationship between appointment non-adherence and greater insurance copays, living in a poverty area [2], lower income [32], and lower socioeconomic class [33]. Participants reported that inadequate patient transportation to appointments and forgetfulness were barriers to healthcare; these relationships have also been corroborated elsewhere [34]–[36].

Our findings indicated that poor hypertension knowledge, not finishing high school, and experiencing side effects were associated with appointment non-adherence. Some authors have previously expressed concern regarding the increasing complexity of appointments scheduling systems, as this may potentially disempower individuals with literacy difficulties or differences in cultural background [17]. These findings highlight the importance of patient education, simple appointment scheduling systems, and good patient-doctor communication. Enhanced and sustained implementation of policies and interventions that address these issues may be useful in reducing the prevalence of appointment non-adherence.

Notably, and in contrast to findings in a study carried out in a similar population [21], this study demonstrated that medication non-adherence was associated with appointment non-adherence. This difference may be explained by differences in study population characteristics including gender, literacy level, and severity of disease. Adherence to appointments is widely used as a marker for medication adherence in clinical practice and research settings despite a lack of clear scientific evidence to support its use [37]–[40]. Our study findings suggests that this practice may not be entirely inappropriate, and among African-Americans with severe, poorly controlled hypertension, measures of medication adherence and appointment attendance are related, even after accounting for confounding factors. Further research is needed to fully elucidate the correlation between these adherence measures.

Interventions such as mail and phone reminders, sms/text messaging, patient education, incentives for keeping appointments, and shared visits have been utilized in reducing appointment non-adherence [17]–[20]. Despite their availability, appointment non-adherence still remains a major burden to the provision of high quality care. In a previous study, a major proportion of the benefit from an intervention designed to improve appointment adherence was obtained from less than one-quarter of patients who had a high risk of missing appointments [41]. This suggests that enhanced targeting of high risk individuals may maximize the impact of interventions.

This study has important limitations. First, these data was focused on urban African-Americans with severe, poorly controlled hypertension, and are not representative of the entire United States population. Secondly, self-reported adherence measures were utilized, which may overestimate adherence compared to objective measurement systems. In addition, due to the inherent limitations of a cross-sectional study design, temporal trends could not be established and causal associations are not inferred.

The number of insured individuals in the United States is expected to markedly increase with the implementation of the Affordable Care Act [42]–[44]. This should mitigate the impact of financial barriers on appointment adherence. However, increased insurance coverage alone will not be sufficient. Providers will need to adapt to rising demands in healthcare by improving patient capacity and clinic efficiency through means such as the optimization of appointment attendance. This study highlights the need for a multi-dimensional framework incorporating relevant factors that provide opportunities for the design of socially appropriate interventions, as well as strengthening existing ones by tailoring them to the peculiar needs of culturally diverse medical populations. Further research is still needed regarding the cost-effectiveness of different interventions in various unique populations.
